# A Model to Predict Prognosis of Renal Cell Clear Cell Carcinoma Based on 3 Angiogenesis-related Long Non-coding RNAs

**DOI:** 10.7150/jca.94685

**Published:** 2024-04-29

**Authors:** Guo Chen, Tiansheng Zhang, Feng Li, Chi Cui, Zhiyong Huang, Xin Gou, Yajun Song, Yang Li

**Affiliations:** 1 Department of Urology, The First Affiliated Hospital of Chongqing Medical University, Chongqing, 400042, China.; 2 Department of Urology, Mianyang Central Hospital, University of Electronic Science and Technology of China, Sichuan Province, 621099, China.; 3 Department of Urology, Three Gorges Hospital of Chongqing University, Chongqing, 404031, China.; 4 Department of General Surgery, The Third People's Hospital of Chengdu, Sichuan Province, 610014, China.; 5 Department of Vascular Surgery, Yibin First People's Hospital, Sichuan Province, 644000, China.; 6 Department of Urology, Xinqiao Hospital of the Army Medical University, Chongqing, 400037, China.

**Keywords:** model, prognosis, renal cell clear cell carcinoma, angiogenesis, lncRNA

## Abstract

**Background:** Tumor angiogenesis is closely related to the progression of clear cell renal cell carcinoma (ccRCC). Long non-coding RNAs (lncRNAs) regulating angiogenesis could be potential biomarkers for predicting ccRCC prognosis. With this study, we aimed to construct a prognostic model based on lncRNAs and explore its underlying mechanisms.

**Methods:** RNA data and clinical information were obtained from The Cancer Genome Atlas (TCGA) database. Angiogenesis-related genes (ARGs) were extracted from the Molecular Signatures database. Pearson correlation and LASSO and COX regression analyses were performed to identify survival-related AR-lncRNAs (sAR-lncRNAs) and construct a prognostic model. The predictive power of the prognostic model was verified according to Kaplan‒Meier curve, receiver operating characteristic (ROC) curve and nomogram analyses. The correlation between the prognostic model and clinicopathological characteristics was assessed via univariate and multivariate analyses. Kyoto Encyclopedia of Genes and Genomes (KEGG) enrichment analysis was subsequently performed to elucidate the mechanisms of the sAR-lncRNAs. In vitro qPCR, immunohistochemistry, migration and invasion assays were conducted to confirm the angiogenic function of sAR-lncRNAs.

**Results:** Three sAR-lncRNAs were used to construct a prognostic model. The model was moderately accurate in predicting 1- , 3- and 5-year ccRCC prognosis, and the risk score according to this model was closely related to clinicopathological characteristics such as T grade and T stage. A nomogram was constructed to precisely estimate the overall survival of ccRCC patients. KEGG enrichment analysis indicated that the MAPK and Notch pathways were highly enriched in high-risk patients. Additionally, we found that the expression of the lncRNAs AC005324.4 and AC104964.4 in the prognostic model was lower in ccRCC cell lines and cancer tissues than in the HK-2 cell line and paracancerous tissues, while the expression of the lncRNA AC087482.1 showed the opposite trend. In a coculture model, knockdown of lncRNA AC005324.4 and lncRNA AC104964.4 significantly promoted the migration and invasion of human umbilical vein endothelial cells (HUVECs), but siR-AC087482.1 transfection alleviated these effects.

**Conclusions:** We constructed a prognostic model based on 3 sAR-lncRNAs and validated its value in clinicopathological characteristics and prognostic prediction of ccRCC patients, providing a new perspective for ccRCC treatment decision making.

## Introduction

Renal cell carcinoma (RCC) is one of the most common malignant urologic tumors worldwide, accounting for more than 430,000 cases and 180,000 deaths annually 1, 2. Clear cell renal cell carcinoma (ccRCC) is the main histopathological subtype and has a high mortality rate 3. Owing to its nonobvious clinical presentation, ccRCC is not often diagnosed before it reaches an advanced metastatic stage 4. Advanced ccRCC is not sensitive to chemotherapy or radiotherapy, resulting in a 5-year overall survival rate of only 10% 5,6. Even with radical nephrectomy, almost 40% of advanced ccRCC cases exhibit recurrence or metastasis 7,8.

Angiogenesis is a dynamic process of neovascularization from capillaries or postcapillary veins and is responsible for tumor metastasis 9. Because of hypoxia in the tumor microenvironment, cancer cells produce a series of proangiogenic factors and provoke unstoppable angiogenesis. These biological activities supply plentiful oxygen and nutrients for rapid tumor growth. Advanced ccRCC is an angiogenesis-dependent tumor 10,11. Therefore, angiogenesis-related biomarkers might be useful for assessing ccRCC prognosis.

The number of specific biomarkers for predicting ccRCC prognosis is limited due to tumor heterogeneity and pathological changes. However, epigenetics, which is characterized by phenotypic changes in gene expression without DNA alterations, provides an optimal scheme for detecting new biomarkers in ccRCC 12-14. Recently, a number of epigenome modifiers and chromatin remodelers have been revealed from genome-wide sequencing of ccRCC. For example, GATA binding protein 5 (GATA5), a DNA-binding transcription factor, is related to poor survival in ccRCC patients 15. MicroRNA-221 and microRNA-32 serve as biomarkers for RCC mortality 16,17. To our knowledge, long non-coding RNAs (lncRNAs) are also involved in epigenetic regulation and are regarded as biomarkers for ccRCC 18.

LncRNAs are transcripts containing more than 200 nucleotides and are not translated into proteins. They participate in multiple crucial physiological and pathological activities, including angiogenesis 19-21. According to previous studies, the combination of the lncRNA LEENE with LEO1 and MYC promoted the transcription of angiogenesis-related genes in a diabetes model 22. The lncRNA LINC00607 induced VEGFA-mediated angiogenic sprouting in endothelial cells 23. Therefore, angiogenesis-related lncRNAs (AR-lncRNAs) can be potential biomarkers for ccRCC prognosis.

**Figure 1:**
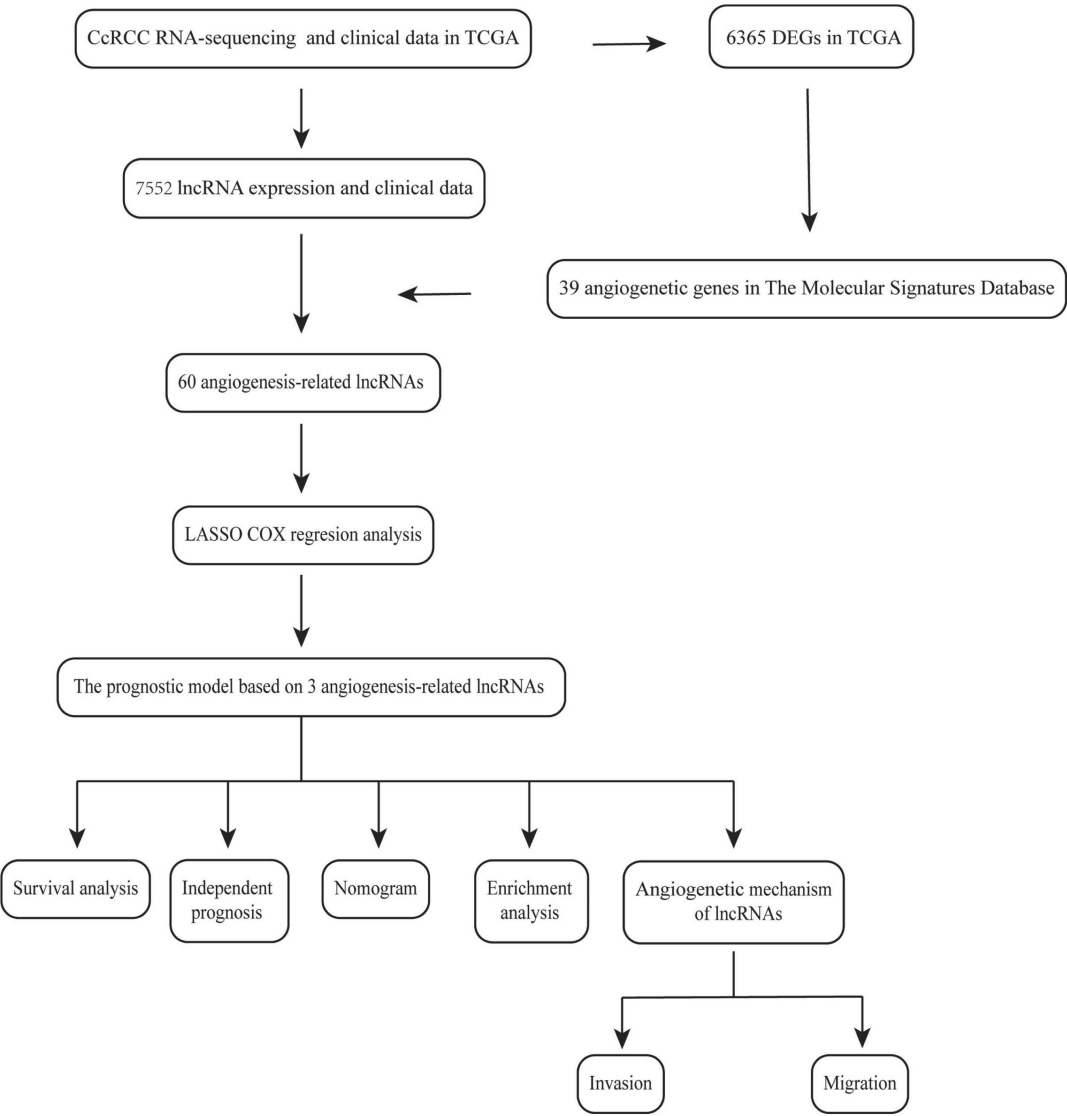
The flow chart of current study.

Herein, we constructed a new model based on 3 AR-lncRNAs to predict ccRCC prognosis. Its association with different clinicopathological characteristics has been well validated. Functional enrichment analysis confirmed that this model was closely related to angiogenetic pathways. Finally, a nomogram was constructed to provide a reference for clinical treatment (Figure 1).

## Materials and Methods

### Data acquisition and download

Transcriptome RNA-sequencing data and clinical information of 531 ccRCC tumor tissue and 72 paracancerous tissue data were downloaded from The Cancer Genome Atlas (TCGA) database (https://portal.gdc.cancer.gov). We excluded patients with incomplete data or unclear survival status. The collected clinical characteristics included overall survival, age, sex, grade, stage, tumor size, distant metastasis and lymph node metastasis. Differentially expressed genes (DEGs) between tumor and paracancerous tissues were identified by screening using differential expression analysis with the R package “LIMMA”. Angiogenesis-related genes (ARGs) were extracted from The Molecular Signatures Database gene sets ANGIOGENESIS-M14493 and HALLMARK_ ANGIOGENESIS (http://www. broad institute. org/gsea/msigdb/index.Jsp).

### Identification of survival AR-lncRNAs (sAR-lncRNAs)

Angiogenesis-related DEGs were identified intersecting DEGs and ARGs with a Venn diagram generated via VENN-4 (http://bioinformatics.psb.ugent.be/webtools/Venn/). Pearson correlation analysis was performed to identify the relationships between glycolysis-related DEGs and lncRNAs. The cutoff criteria of |r| > 0.4 and *p* < 0.05 were used to determine the AR-lncRNAs. Potential sAR-lncRNAs were selected using univariate COX analysis based on the “survival” R package. These sAR-lncRNAs were further divided into risk-related and protective factor groups according to the hazard ratios (HRs) (positive vs. negative).

### Construction of a prognostic model based on sAR-lncRNAs

Through LASSO and COX regression analyses, the prognostic model was constructed by multiplying the expression levels of the 3 sAR-lncRNAs. The formula for the angiogenesis-related prognostic model was [expression level of the lncRNA AC087482.1 × (0.146544)] + [expression level of the lncRNA AC104964.4 × (-0.0244075)] + [expression level of the lncRNA AC005324.4 × (-0.0466575)].

### Evaluation of the prognostic model

Kaplan-Meier curves were generated, and overall survival was compared between the high-risk and low-risk groups via the “maxstat” R package. Receiver operating characteristic (ROC) curves were used to evaluate the sensitivity and specificity of the prognostic model and clinicopathological characteristics. Furthermore, the risk score was verified as an independent prognostic factor for ccRCC patients by univariate and multivariate COX regression analyses.

### Gene set enrichment analysis

Kyoto Encyclopedia of Genes and Genomes (KEGG) enrichment analysis was performed to detect enrichment of pathways between the high-risk and low-risk groups with the R “clusterProfiler” package. A normalized enrichment score (|NES|) >1.5 and *p* < 0.05 indicated a significant difference.

### Analysis of the molecular mechanisms of sAR-lncRNAs

MAPK-related genes (MRGs) and Notch pathway-related genes (NRGs) pathways were obtained from the PathCards database (https://pathcards.genecards.org/). MAPK/Notch-related DEGs were identified in VENN-4 by taking the intersection of DEGs, MRGs and NRGs. After Pearson correlation analysis of MAPK/Notch-related DEGs and the risk score, sAR-lncRNA-related MRGs and NRGs (lncRNA-genes) were identified (|r| > 0.4 and p < 0.05). A protein interaction network was constructed using the Search Tool for the Retrieval of Interacting Genes/Proteins (STRING) database to investigate the interactions between angiogenic proteins and proteins encoded by lncRNA-genes (lncRNA-proteins).

### Establishment and validation of the nomogram

We utilized the “rms” R package to establish a nomogram by integrating clinical features, the risk score and overall survival, which was a useful tool for clinical prognosis prediction in ccRCC patients. Next, 1-, 3- or 5-year calibration curves and ROC curves were used to validate the efficiency of the nomogram.

### Clinical samples

From February 2020 to April 2023, a total of 30 ccRCC tissues and paracancerous tissues from patients who underwent surgical resection at the First Affiliated Hospital of Chongqing Medical University were included in this study (Table 1). Patients with severe underlying disease or other cancers were excluded. The lncRNAs were divided into those with high expression of sAR-lncRNAs (hlncRNAs, n=9) and those with low expression of sAR-lncRNAs (llncRNAs, n=21) according to the median expression of lncRNA AC005324.4 and lncRNA AC104964.4. This study was approved by the Medical Ethics Committee of the First Affiliated Hospital of Chongqing Medical University (2020-51). All patients provided informed consent.

### Cell culture

The human proximal tubular epithelial (HK-2) cell line and human ccRCC cell lines (786-O, Caki-1, and RCC-JF) were purchased from Pricella. The cells were cultured in RPMI-1640 (786-O), McCoy's 5A (Caki-1) and DMEM (RCC-JF) media (Gibco, USA) supplemented with 10% fetal calf serum (FBS; Biolnd, Israel), 100 U/mL penicillin (Beyotime, China) and 100 μg/mL streptomycin (Beyotime, China) in a 37 °C incubator under 5% CO2.

### Cell transfection

The siRNAs targeting the lncRNAs AC087482.1, AC005324.4 and AC104964.4 were used to silence the expression of the corresponding lncRNAs. The sequences are shown in Table 2. CcRCC 786-O cells were cultivated in a 6 cm petri dish. When the cells reached 60%-70% confluence, siRNAs (10 μL, 20 nM) were transfected into 786-O cells with 5 μL of Lipofectamine 3000 (Invitrogen, USA) for 8 hours. Then, the culture medium was replaced with fresh culture medium, and the cells were cultivated for 24 h. qPCR was used to validate the effects of the siRNAs.

### RT‒qPCR

RNA from cells and tissues was extracted by TRIzol Reagent (ABclonal, China) and reversed transcribed to cDNA by ABScript III RT Master Mix for qPCR with gDNA remover (ABclonal, China). qPCR was performed using SYBR Green Fast qPCR Mix (ABclonal, China) on a 7500 Real-Time PCR System (Thermo Fisher, USA). Gene expression was calculated by the 2^-ΔΔCT^ method and normalized according to the expression of β-actin. The primer sequences are shown in Table 2.

### Western blotting

Proteins from cells were extracted with RIPA lysis buffer (Beyotime, China) supplemented with protease inhibitors (Beyotime, China). Proteins were separated through sodium dodecyl sulfate‒polyacrylamide gel electrophoresis (SDS‒PAGE) and then bolted on polyvinylidene difluoride (PVDF) membranes (Millipore, USA). After blocking nonspecific sites with 5% milk (Beyotime, China) for 1 hour, the PVDF membranes were incubated with primary antibodies against CD31, VEGFA, N-cadherin and vimentin (Proteintech, China) at 4 °C overnight. The membranes were then incubated with secondary antibodies (Bioss, China) at room temperature for 1 hour. The protein bands were visualized using a Fusion FX System (Vilber, Germany).

### Immunohistochemistry (IHC)

The tissue was fixed in 4% buffered formalin (Biosharp, China). Following dehydration, antigen retrieval and blocking, the tissue was incubated with primary antibodies against CD31 and VEGFA (Proteintech, China) at 4 °C overnight. The membranes were then incubated with secondary antibodies (Bioss, China) at room temperature for 1 hour. The sections were stained with diaminobenzidine (DAB, Sigma-Aldrich, Germany) and hematoxylin. The number of DAB-positive cells in 5 random fields was assessed under an optical microscope (Olympus, Japan).

### Transwell assay

For cell migration assays, 500 μL of conditioned medium from siRNA-treated cells was added to the lower chamber (Corning, USA), and 3×10^5^ human umbilical vein endothelial cells (HUVECs) without FBS were seeded into the upper chamber. For cell invasion assays, Matrigel was diluted with RPMI-1640 and evenly spread on the bottom of the upper chamber. After the Matrigel solidified, 3×10^5^ HUVECs in 100 μl of RPMI-1640 basal medium were added to the upper chamber, while 600 μL of conditioned medium from the siRNA cells was added to the lower chamber. After 24 hours and 48 hours of incubation for the migration and invasion assays, the transwell inserts were fixed with 4% paraformaldehyde. Finally, the inserts were dyed with 0.1% crystal violet solution for 20 min, and the invading cells were observed under an optical microscope (Olympus, Japan).

### Statistical analysis

The data were analyzed using R software (version 4.1.0), SPSS 26.0, and GraphPad Prism 8.4. The PERL programming language (version 5.30.2) was used to process the data. Pearson's correlation coefficient was used to assess linear correlations. ROC curves were generated to predict the model accuracy. COX regression was used to identify factors that could independently predict overall survival. LASSO analysis was used to perform screening of the AR-lncRNAs. Continuous data and categorical data were analyzed by Student's test and the chi-square test. *p*< 0.05 was considered to indicate a statistically significant difference.

## Results

### Acquirement of AR-lncRNAs

**Figure 2:**
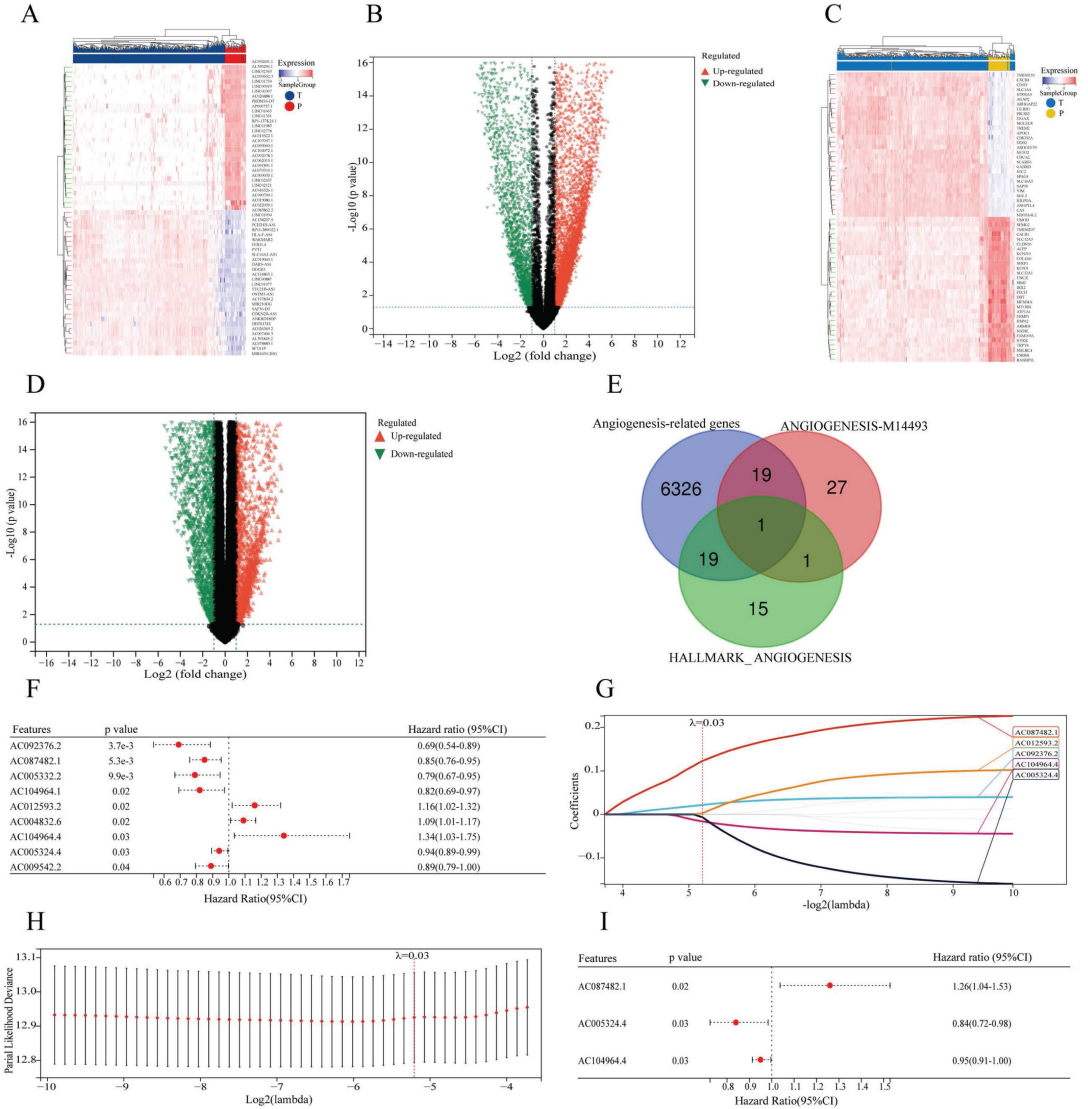
Identification of AR-lncRNAs. (A-D) Heatmaps and volcano plots showing the differentially expressed lncRNAs (A, B) or genes (C, D) between ccRCC tissues and paracancerous tissues in the TCGA. In the heatmap, the red parts represent upregulated lncRNAs/genes, and the blue parts represent downregulated lncRNAs/genes. In the volcano plot, the green dots represent downregulated lncRNAs/genes, the red dots represent upregulated lncRNAs/genes, and the black dots represent lncRNAs/genes with no differential expression (log2 |FC| > 1, p < 0.05). (E) Venn diagram illustrating 39 angiogenesis-related genes identified from the TCGA, HALLMARK and GSEA databases. (F) Forest plot showing the 9 prognostic differentially expressed sAR-lncRNAs according to univariate COX regression analysis. (G) LASSO regression analysis was carried out to identify 5 sAR-lncRNAs. (H) The optimal LASSO model was constructed with the best parameter (λ=0.03). (I) Forest plot showing the 3 prognostic differentially expressed sAR-lncRNAs according to multivariate COX regression analysis. AR-lncRNAs, angiogenesis-related lncRNAs; ccRCC, clear cell renal cell carcinoma; TCGA, The Cancer Genome Atlas; sAR-lncRNAs, survival AR-lncRNAs.

The DEGs in 531 ccRCC tissues and 72 paracancerous tissues were extracted from the TCGA database, and 7552 lncRNAs and 6365 mRNAs were identified (Figure 2A-D). By intersecting the selected mRNAs and two angiogenesis-related gene sets (Table S1), a total of 39 angiogenesis-related differentially expressed mRNAs were identified in the TCGA cohort (Figure 2E). By using Pearson correlation analysis between differentially expressed lncRNAs and angiogenesis-related mRNAs, 60 lncRNAs associated with angiogenesis were identified. After univariate COX analysis, 9 angiogenesis-related lncRNAs related to overall survival were identified (Figure 2F). Finally, by LASSO and multivariate COX regression analysis, 3 angiogenesis-related lncRNAs, AC087482.1, AC104964.4 and AC005324.4, were identified as candidate independent prognostic factors for the prognostic model (Figure 2G-I).

### Establishment of the sAR-lncRNA prognostic model

**Figure 3:**
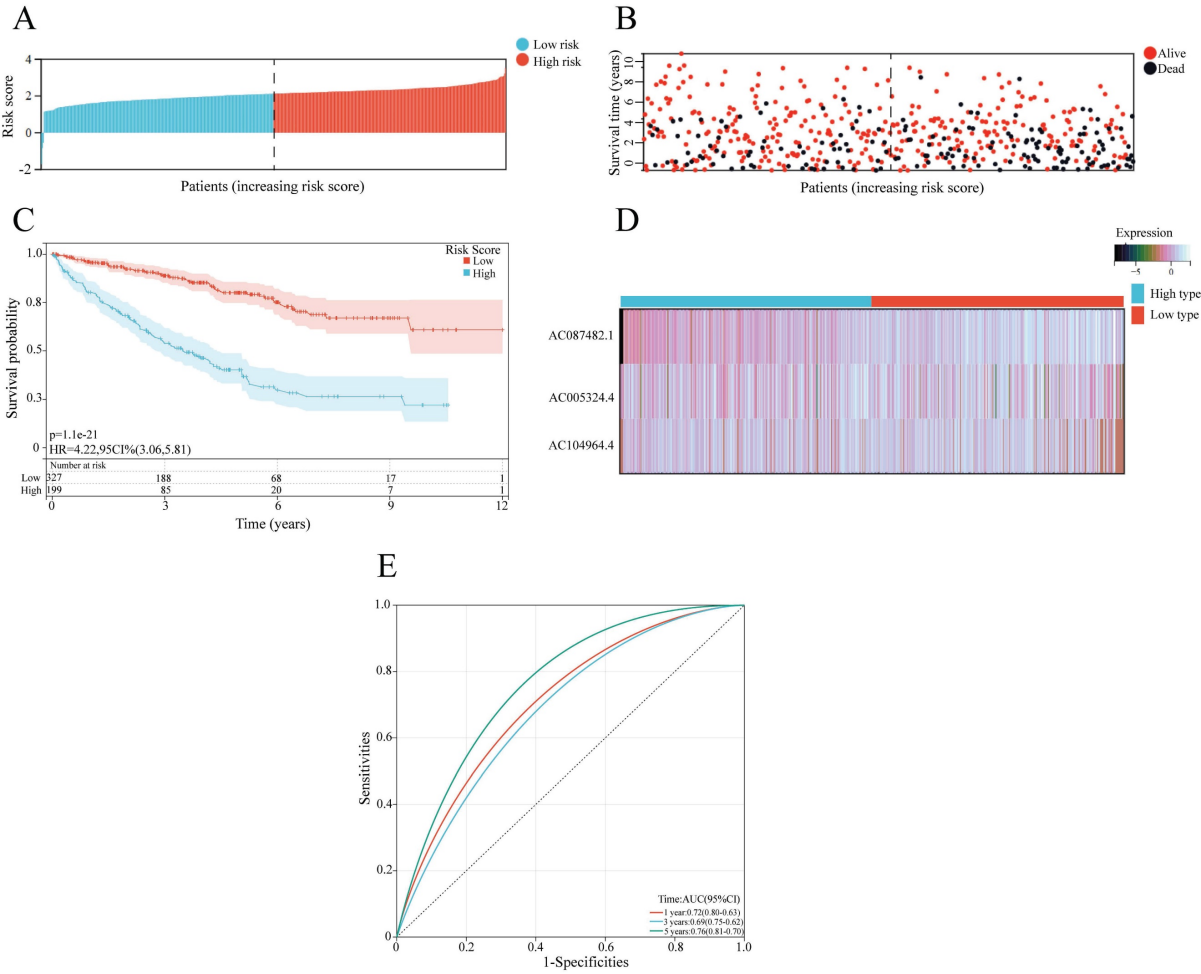
Construction of a prognostic model based on sAR-lncRNAs. (A, B) Risk score distribution (A) and survival status of patients (B) in the high-risk and low-risk groups. (C) Kaplan‒Meier survival curve analysis showing the difference in survival time between the low-risk and high-risk groups. (D) Heatmap showing the expression of 3 sAR-lncRNAs between the high-risk and low-risk groups. (E) ROC curves showing the AUC values of the prognostic model (AUC 1 year=0.72, AUC 3 years=0.69, AUC 5 years=0.76). sAR-lncRNAs, survival AR-lncRNAs

A prognostic model based on 3 angiogenesis-related lncRNAs was constructed for evaluating the prognosis of ccRCC patients. According to the mean risk score, ccRCC patients were classified into a high-risk group and a low-risk group (Figure 3A). The number of surviving patients with ccRCC decreased as the risk score increased, indicating that a high risk sore was correlated with a worse prognosis (Figure 3B, C). For the AR-lncRNAs, the expression of the lncRNA AC087482.1 was a prognostic risk factor that increased with increasing risk score, while the expression of the lncRNAs AC104964.4 and AC005324.4 decreased as the risk score increased, indicating that these lncRNAs are protective factors (Figure 3D). Moreover, the ROC curves showed that the AUCs were 0.72, 0.69 and 0.76 at 1, 3, and 5 years, respectively, which confirmed the sensitivity and specificity of the model for accurately predicting patient prognosis (Figure 3E).

### Correlation analyses of the sAR-lncRNA prognostic model and clinicopathological characteristics

**Figure 4:**
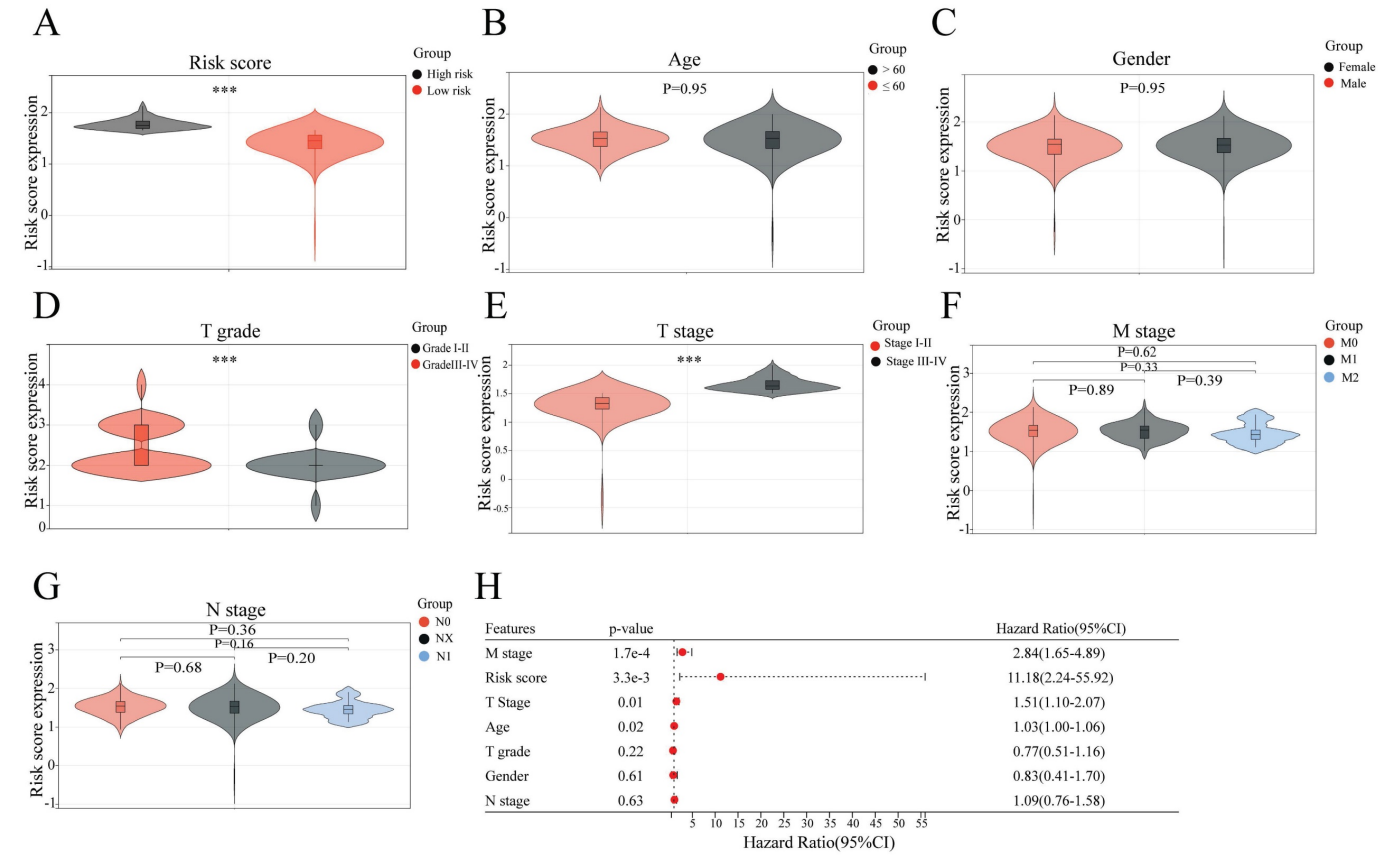
Correlation analysis of the sAR-lncRNA prognostic model risk score and clinicopathological characteristics. (A-G) The risk score according to the prognostic model is closely related to clinicopathological characteristics; the risk score (A) (high risk vs. low risk), T grade (D) (grade I-II vs. grade III-IV), and T stage (E) (stage I-II vs. stage III-IV). Age (B), gender (C), M stage (F) and N stage (G) were not different between groups. (H) Forest plot showing the risk score and other clinical features related to overall survival according to multivariate COX regression analysis. ***p <0.01. sAR-lncRNAs, survival AR-lncRNAs.

To confirm the association of the risk score of the sAR-lncRNA prognostic model with clinicopathological characteristics, correlation analyses were conducted to compare the clinicopathological characteristics between the high- and low-risk groups. The results showed that T grade (*p* < 0.01), T stage (*p* < 0.01) and risk score (*p* < 0.01) were significantly different between the two groups, while N stage (*p* = 0.36), M stage (*p* = 0.62), gender (*p* = 0.95) and age (*p* = 0.95) were not (Figure 4A-G). After multivariate COX regression analysis, clinicopathological characteristics such as risk score, age, M stage and T stage were identified as independent risk factors for ccRCC prognosis (Figure 4H). The above results proved that the risk score according to the sAR-lncRNA prognostic model is significantly correlated with clinicopathological characteristics in ccRCC patients.

### Construction of the nomogram

**Figure 5:**
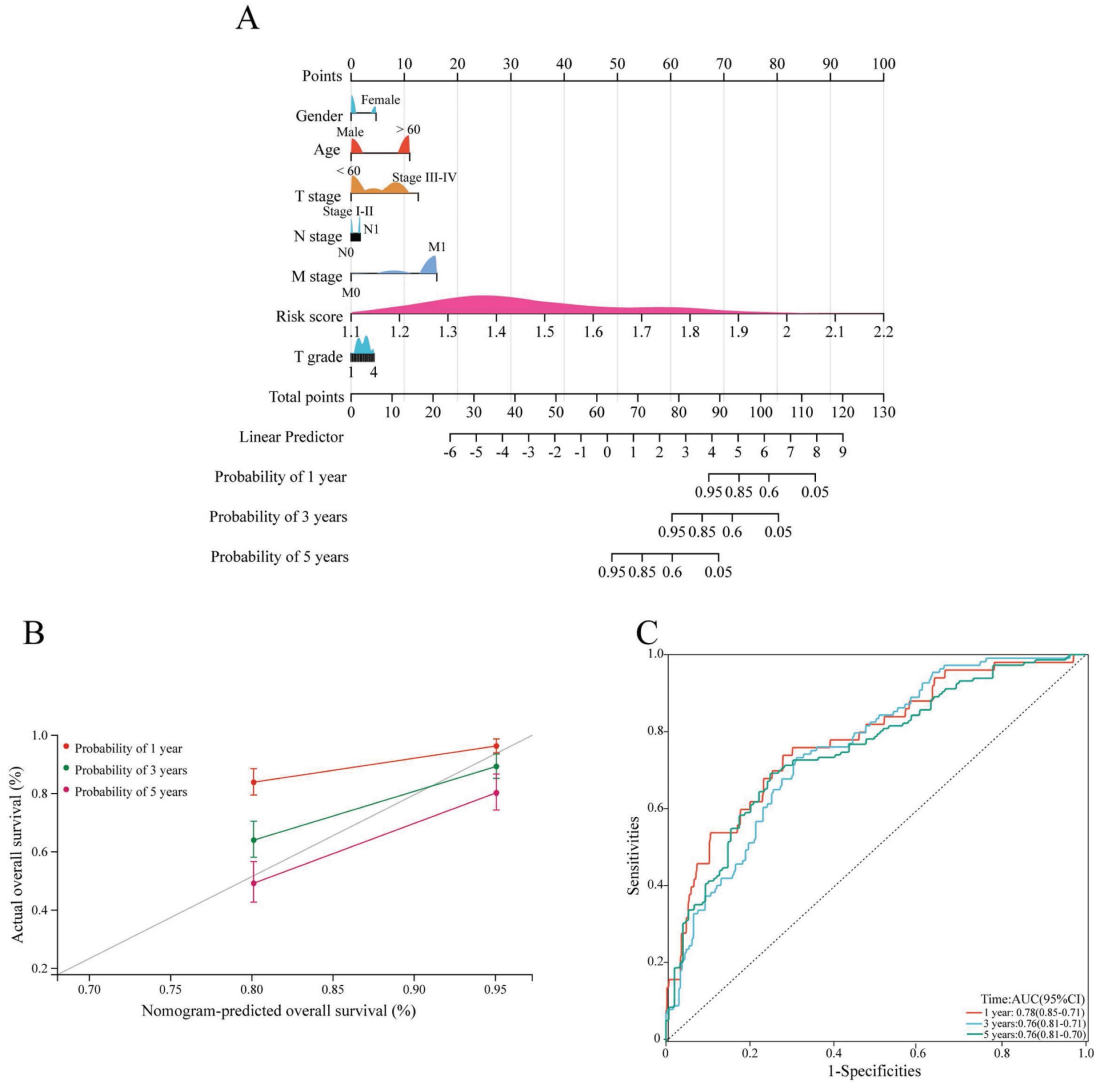
Construction and validation of the nomogram. (A) The nomogram shows the 1-, 3-, and 5-year survival probability of ccRCC patients according to the risk score and clinical characteristics. (B) Calibration curves revealing the concordance between the predicted and observed overall survival of ccRCC patients at 1, 3 and 5 years. (C) ROC curves showing the AUC values of the nomogram (AUC 1 year=0.78, AUC 3 years=0.76, AUC 5 years=0.76). ccRCC, clear cell renal cell carcinoma.

Next, a nomogram based on clinicopathological characteristics and the risk score was constructed to estimate the 1-, 3- and 5-year overall survival probabilities of ccRCC patients. To assess the overall survival probability, the risk score for each patient was determined by adding the score for each prognostic variable (Figure 5A). The calibration curve demonstrated that the predicted overall survival was consistent with the actual survival rate at 1, 3, and 5 years (Figure 5B). The AUC values of the ROC curves were 0.78, 0.76 and 0.76 at 1, 3, and 5 years, respectively (Figure 5C).

### Functional enrichment analysis

**Figure 6:**
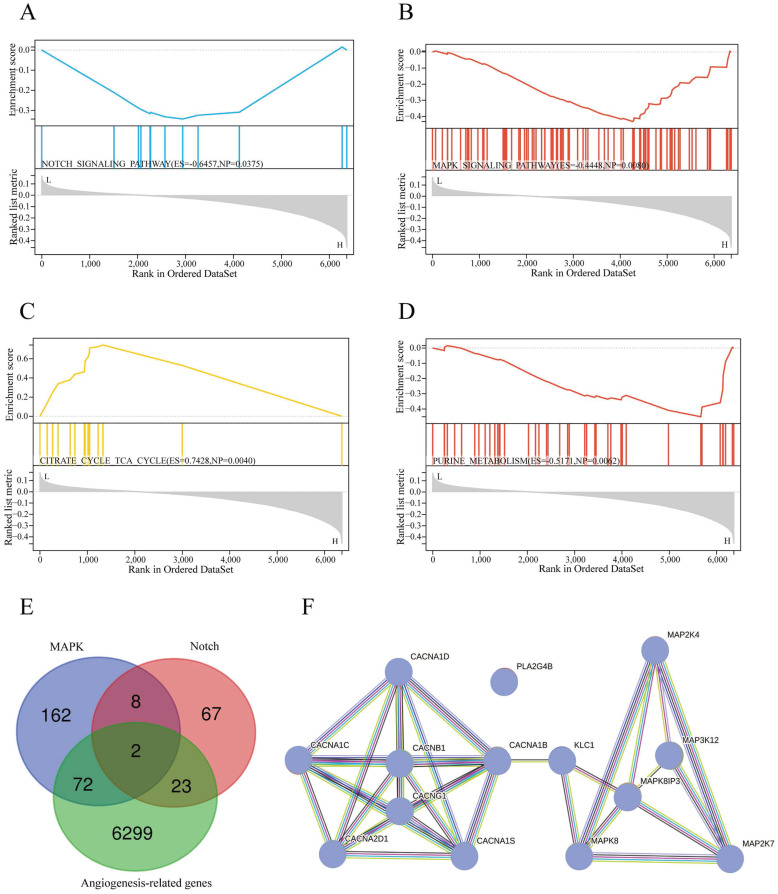
Functional enrichment and molecular mechanism analysis. (A-D) KEGG pathway analysis showing the significantly enriched pathways in the high-risk group, such as NOTCH signaling (A) (ES=-0.6457, NP=0.0375), MAPK signaling (B) (ES=-0.4448, NP=0.008), the citrate cycle TCA cycle (C) (ES=0.7428, NP=0.004), and purine metabolism (D) (ES=-0.5171, NP=0.0062). (E) Venn diagram illustrating 74 MAPK-related DEGs and 25 Notch-related DEGs identified by screening of DEGs, MRGs and NRGs. (F) Protein interaction network of lncRNAs-proteins. TCGA, The Cancer Genome Atlas; DEGs: differentially expressed genes; MRGs: MAPK-related genes; NRGs: Notch pathway-related genes; lncRNA-proteins: proteins translated by lncRNA-genes.

To determine the signaling pathways of sAR-lncRNAs in the prognostic model. The KEGG database used to explore the differences in the enrichment of signaling pathways between the high-risk and low-risk groups. Pathways such as the Notch pathway (Figure 6A), MAPK pathway (Figure 6B), citrate cycle, TCA cycle (Figure 6C) and purine metabolism (Figure 6D) were activated in the high-risk group.

### Analysis of the molecular mechanisms of sAR-lncRNAs

To further explore the molecular mechanisms of sAR-lncRNAs in the prognostic model, a total of 74 MAPK-related DEGs and 25 Notch-related DEGs were identified by taking the intersection of the DEGs, MRGs and NRGs (Figure 6E). By Pearson correlation analysis of MAPK/Notch-related DEGs and the risk score, a total of 4 lncRNA-genes, PLA2G4B, MAP3K12, MAPK8IP3 and CACNB1, were identified as key genes correlated with sAR-lncRNAs in the prognostic model. The results of the protein interaction network analysis revealed that 10 proteins could interact with the lncRNA-proteins (Figure 6F).

### The levels of the lncRNAs AC005324.4 and AC104964.4 are decreased, and the level of the lncRNA AC087482.1 is increased in ccRCC tissues and cell lines

**Figure 7:**
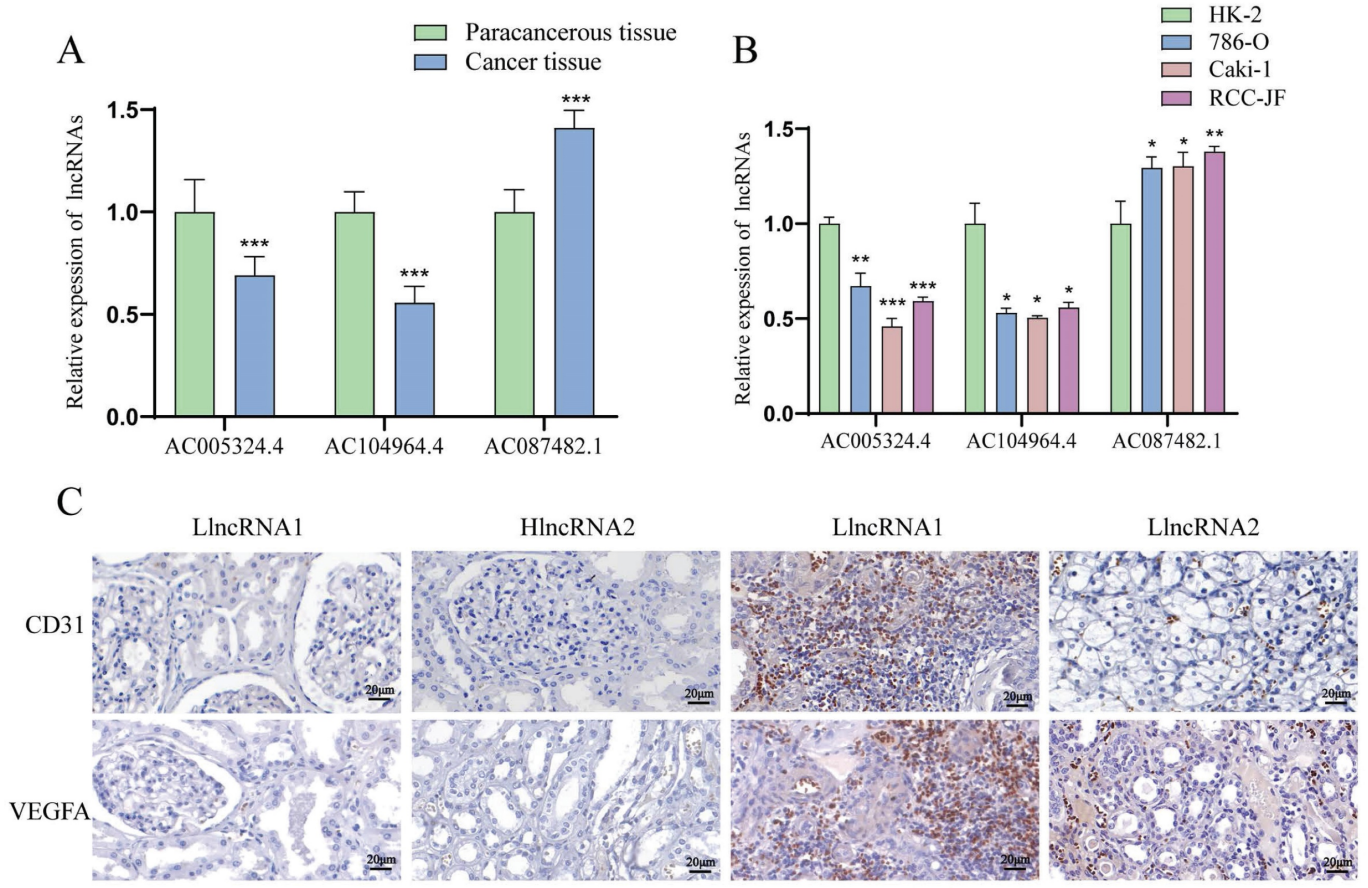
The expression of AC005324.4, AC104964.4 and AC087482.1 in cell lines and clinical tissues. (A, B) qPCR results showing the expression of AC005324.4, AC104964.4 and AC087482.1 in clinical tissues (A) (30 cancer tissues and 30 paracancerous tissues) and cell lines (B) (HK-2, 786-O, Caki-1 and RCC-JF, n=3/group); the lncRNA levels in the cancer group were normalized according to the levels in the paracancerous tissue/HK-2 group; one-way ANOVA followed by Tukey's test. (C) IHC shows the percentages of cells with VEGFA and CD31 staining in the llncRNA group and hlncRNA group; scale bar: 20 μm. *p <0.5, **p <0.01, ***p <0.001. IHC, immunohistochemistry; llncRNA, low expression of sAR-lncRNA; hlncRNA, high expression of sAR-lncRNA.

LncRNA AC005324.4 and lncRNA AC104964.4 were downregulated in cancer tissues (n=30), while lncRNA AC087482.1 was significantly upregulated (Figure 7A). Compared to those in the HK-2 cell line, the expression levels of the lncRNAs AC005324.4 and AC104964.4 were lower in the 786-O, Caki-1 and RCC-JF cell lines (n=3), while the opposite trend was observed for the lncRNA AC087482.1 (Figure 7B). In addition, the CD31 and VEGFA protein levels were lower in the hlncRNA group (n=9), indicating that the lncRNAs AC005324.4 and AC104964.4 suppress the generation of endothelial cells in ccRCC (Figure 7C).

### LncRNA AC005324.4 and lncRNA AC104964.4 inhibit the migration and invasion of HUVECs, while lncRNA AC087482.1 has the opposite effect

**Figure 8:**
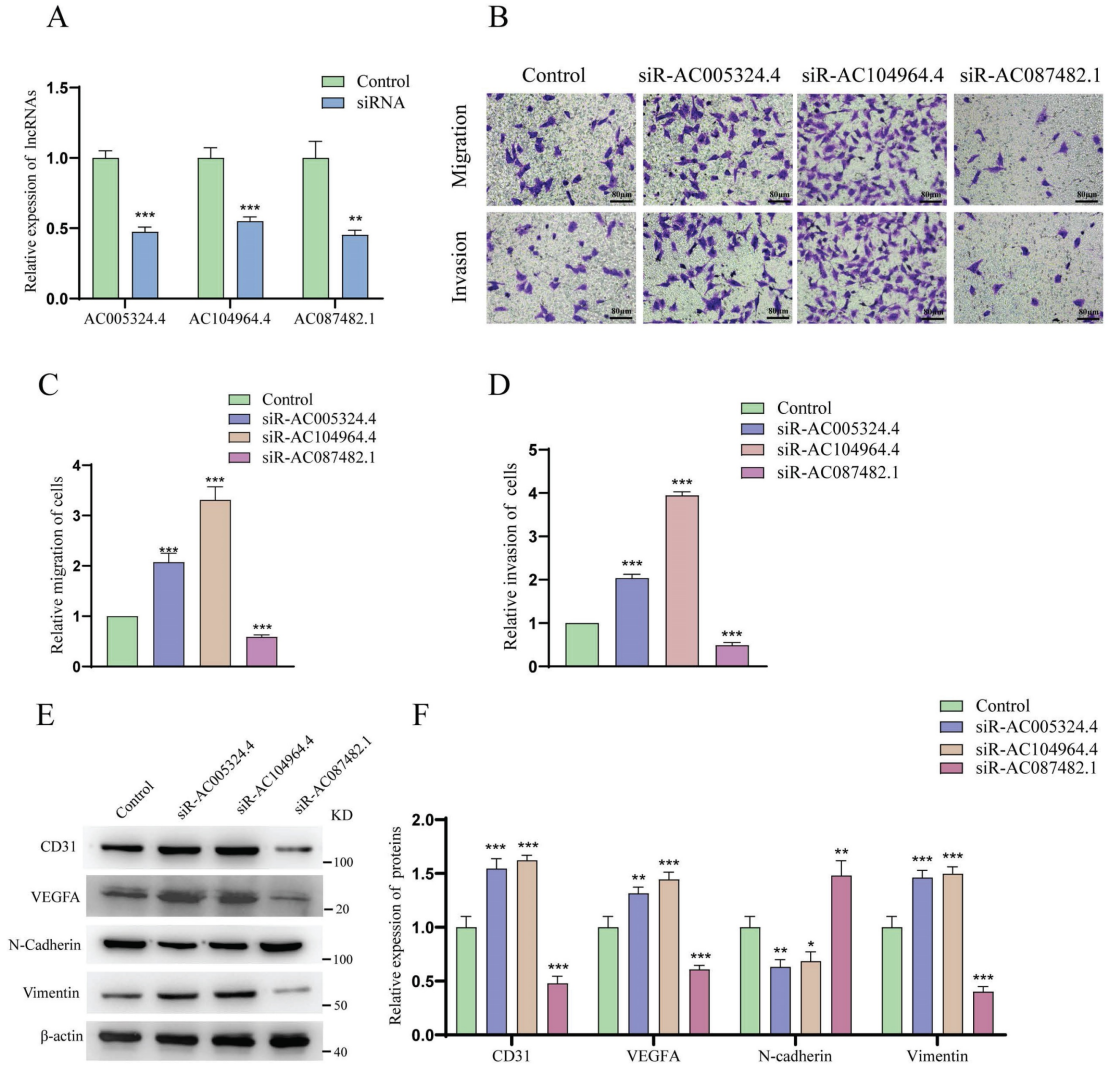
The effects of AC005324.4, AC104964.4 and AC087482.1 on invasion and migration. (A) qPCR confirmed the effects of siR-AC005324.4, siR-AC104964.4 and siR-AC087482.1 in 786-O cells (n=3/group); the levels in the experimental groups were normalized to those of the control group; one-way ANOVA followed by Tukey's test. (B-D) The migration and invasion status of HUVECs in the control, siR-AC005324.4, siR-AC104964.4 and siR-AC087482.1 groups (B) (scale bar: 80 μm), and via quantitative analysis (C, D) (n=3/group); the experimental group cells were used for normalization; one-way ANOVA followed by Tukey's test. (E, F) The protein levels of CD31, VEGF-A, N-cadherin and vimentin were measured in the control, siR-AC005324.4, siR-AC104964.4 and siR-AC087482.1 groups (E) and via quantitative analysis (F) (n=3/group); the levels in the experimental groups were normalized to the levels in the control group; one-way ANOVA followed by Tukey's test. *p <0.5, **p <0.01, ***p <0.001.

As shown in Figure 8A, the effects of siR-AC005324.4, siR-AC104964.4 and siR-AC087482.1 were confirmed in 786-O cells (n=3). The migration and invasion of HUVECs cocultured with siR-AC005324.4 or siR-AC104964.4 786-O cells were more obvious than those of the control group, but the migration and invasion of those cocultured with siR-AC087482.1 786-O cells were suppressed (Figure 8B-D). In the protein expression analysis, CD31, VEGFA and vimentin levels increased, while N-cadherin decreased in siR-AC005324.4 and siR-AC104964.4 cells, which was contrary to the findings in siR-AC087482.1 cells (Figure 8E, F). These findings indicated that lncRNA AC005324.4, lncRNA AC104964.4 and lncRNA AC087482.1 regulate angiogenesis in ccRCC by mediating the invasion and migration of HUVECs.

## Discussion

Advanced ccRCC is characterized by high heterogeneity and easily metastasizes, so advanced ccRCC patients have a very poor prognosis. Potential biomarkers such as lncRNAs play a significant role in guiding treatment decision making. Compared to single lncRNAs, a model constructed with representative lncRNAs may perform better for ccRCC prognosis evaluation 24. Compared to existing signatures including several lncRNAs (more than 6) 25, 26, our 3-lncRNA model contains the fewer genes and should be easy to apply in the clinic. Different functional lncRNA models, specifically ferroptosis/cuproptosis-associated lncRNA signatures and tumor-infiltrating lymphocyte-related lncRNA models, have been reported to predict ccRCC prognosis 27-29. However, these lncRNAs in models are closely related to immunotherapy and drug response but not to ccRCC prognosis. Notably, the growth of advanced ccRCC is highly dependent on the blood supply, and angiogenesis significantly impacts the metastasis risk, recurrence risk and prognosis of ccRCC patients 30,31. Therefore, a prognostic model constructed with AR-lncRNAs could be an optimal model with good prognostic value and predictive performance for ccRCC.

In the present study, we first established a novel prognostic model based on 3 sAR-lncRNAs, namely, lncRNA AC087482.1, lncRNA AC005324.4 and lncRNA AC104964.4. This model performed well in predicting the overall survival and clinicopathological characteristics of ccRCC patients. In addition, the sAR-lncRNA model-based nomogram could be used to easily calculate scores, which could assist in personalized treatment.

We also noted that high-risk ccRCC samples exhibited enrichment of genes related to the Notch pathway, MAPK, the citrate cycle and purine metabolism. The Notch pathway has a significant impact on angiogenesis. Ji-Liang et al. reported that the Notch pathway regulates angiogenic processes in endothelial cells 32. Crosstalk between the VEGF and Notch pathways has emerged in tumor angiogenesis: notch signaling alters the expression of receptors (NRP1/2 and VEGFR1/2/3), promoting VEGF signaling 33. The MAPK pathway also regulates tumor angiogenesis. Ramesh et al. reported that FGFR receptors activate Ras/MAPK signaling to promote cell survival, proliferation and angiogenesis 34,35. These findings further proved that the activation of angiogenesis-related pathways is directly related to the poor prognosis of ccRCC. Notably, disturbances in the citrate cycle and purine metabolism are correlated with a poor prognosis in ccRCC patients 36. Thus, targeting angiogenesis, the citrate cycle and purine metabolism are promising strategies for preclinical investigations on improving ccRCC prognosis.

The identification of Notch/MAPK-related genes is important for elucidating the molecular mechanisms involved in high-risk ccRCC. To develop a model with advantages over other reported sAR-lncRNA predictive models 37,38, we first identified 4 potential lncRNA-genes/-proteins associated with ccRCC prognosis. Notably, MAP3K12 and PLA2GB participate in angiogenic processes. Sun et al. reported that MAP3K12 promotes the MEK/ERK/EIF4E/HIF1-α/VEGFA axis by binding to RIT1 in hepatocellular carcinoma 39. Hu et al. reported that PLA2GB is an effective indicator of sphingolipid metabolism, which is involved in the formation of new blood vessels in osteosarcoma metastasis 40. Furthermore, MAP2K4 and MAP2K7 mediate MAPK pathway activity to promote angiogenesis by interacting with MAP3K12. The interaction network revealed the potential molecular mechanisms of sAR-lncRNAs in the model and revealed lncRNA-proteins and interacting proteins that could be prognostic biomarkers or therapeutic targets for ccRCC.

Three sAR-lncRNAs extracted from this prognostic model can be regarded as prognostic markers and therapeutic targets for ccRCC. LncRNA AC104964.4 expression is associated with the sensitivity to docetaxel, cisplatin, and S-1 (DCS) treatment. Yang et al. reported that the expression of the lncRNA AC104964.4 decreased in cisplatin-resistant gastric cancer (GC) cells, suggesting that lncRNA AC104964.4 is involved in the response to chemotherapeutic drugs 41. It is also negatively related to immune infiltration (neutrophils, mast cells and Treg cells), indicating a relatively good prognosis in patients with GC 42. In our study, we found for the first time that lncRNA AC005324.4, lncRNA AC104964.4 and lncRNA AC087482.1 are associated with angiogenesis in ccRCC. High expression of the sAR-lncRNAs in ccRCC tissues promoted neovascular generation by upregulating the CD31 and VEGFA proteins in vivo Moreover, high expression of sAR-lncRNAs increased angiogenesis by stimulating HUVEC migration and invasion in vitro. Therefore, sAR-lncRNAs merit further investigation as antiangiogenic targets.

Notably, our research has some limitations. First, transcriptome data and overall survival information were obtained only from the TCGA dataset, and more datasets are needed to further validate the uniformity of the prognostic model. Second, the number of clinical samples and ccRCC cell lines was small, so more samples are needed to strengthen our results. Finally, the mechanisms of sAR-lncRNAs, including their functions in the citrate cycle and purine metabolism and their interactions with angiogenesis, need to be further clarified. Future studies on the molecular mechanisms of sAR-lncRNAs should include in vivo and in vitro experiments related to the KEGG results.

## Conclusions

In this study, we established a predictive model with 3 sAR-lncRNAs. This model not only identifies a relationship between lncRNAs and angiogenesis but also has high accuracy in ccRCC prognosis prediction. It is a useful tool to assist in ccRCC therapeutic decision making.

## Supplementary Material

Supplementary table.

## Figures and Tables

**Table 1:** Demographic and clinical data of patients with clear cell renal cell carcinoma.

Character	Number
Age (years)	
≤60	18
>60	12
Gender	
Male	20
Female	10
T grade	
I-II	13
III-IV	17
T stage	
I-II	21
III-IV	9
M stage	
Negative	5
Positive	25
N stage	
Negative	3
Positive	27
LlncRNA	21
HlncRNA	9

LlncRNA, low expression of sAR-lncRNAs; hlncRNA, high expression of sAR-lncRNAs

**Table 2:** The siRNA and primer sequences.

Item	Sequence
**Primer**	
LncRNA AC087482.1	Forward CCAACCATTGAGAGGTAGCCA
	Reverse TTACAGCGGAGAGAACGGAC
LncRNA AC005324.4	Forward CCACTACCCCTCCCAGATGA
	Reverse TCCCGTGTGCTCAAATCCTC
LncRNA AC104964.4	Forward CACGGTCATCAGCTACAGGC
	Reverse GCTTGCCAGGGATTGTAGGA
GAPDH	Forward CCTTCCGTGTCCCCACT
	Reverse GCCTGCTTCACCCACCTTC
**siRNA**	
siR- lncRNA AC087482.1	Forward GGAUCCCUGCUCUUCUAUA
	Reverse UAUAGAAGAGCAGGGAUCC
siR- lncRNA AC005324.4	Forward AGUAGGAGACAAGAAUUAA
	Reverse UUAAUUCUUGUCUCCUACU
siR- lncRNA AC104964.4	Forward GGCCUAUUUCAGAUAAGAA
	Reverse UUCUUAUCUGAAAUAGGCC

## Abbreviations

LncRNAs: Long non-coding RNAs
CcRCC: Clear cell renal cell carcinoma
TCGA: The Cancer Genome Atlas
ARGs: Angiogenesis-related genes
sAR-lncRNAs: The survival AR-lncRNAs
ROC: Receiver operating characteristic
KEGG: Kyoto Encyclopedia of Genes and Genomes
MRGs: Genes related to MAPK
NRGs: genes related to Notch pathways
LncRNA-genes: sAR-lncRNAs related MAPK/ Notch genes
LncRNA-proteins: Proteins translated by lncRNA-genes
HUVECs: Human umbilical vein endothelial cells
RCC: Renal cell carcinoma
GATA5: GATA Binding Protein 5
AR-lncRNAs: Angiogenesis-related lncRNAs
DEGs: Differentially expressed genes
HR: Hazard ratio
LlncRNAs: Low expression of sAR-lncRNAs
HlncRNAs: high expression of sAR-lncRNAs
DCS: Docetaxel, cisplatin, and S-1
GC: Gastric cancer

## References

[1] Campi R, Rebez G, Klatte T, Roussel E, Ouizad I, Ingels A (2023). Effect of smoking, hypertension and lifestyle factors on kidney cancer - perspectives for prevention and screening programmes. Nat Rev Urol.

[2] Makino T, Kadomoto S, Izumi K, Mizokami A (2022). Epidemiology and Prevention of Renal Cell Carcinoma. Cancers (Basel).

[3] Jonasch E, Walker CL, Rathmell WK (2021). Clear cell renal cell carcinoma ontogeny and mechanisms of lethality. Nat Rev Nephrol.

[4] Jiao Q, Bi L, Ren Y, Song S, Wang Q, Wang YS (2018). Advances in studies of tyrosine kinase inhibitors and their acquired resistance. Mol Cancer.

[5] Ballesteros PÁ, Chamorro J, Román-Gil MS, Pozas J, Gómez Dos Santos V, Granados ÁR (2021). Molecular Mechanisms of Resistance to Immunotherapy and Antiangiogenic Treatments in Clear Cell Renal Cell Carcinoma. Cancers (Basel).

[6] Dabestani S, Thorstenson A, Lindblad P, Harmenberg U, Ljungberg B, Lundstam S (2016). Renal cell carcinoma recurrences and metastases in primary non-metastatic patients: a population-based study. World J Urol.

[7] Sánchez-Gastaldo A, Kempf E, González Del Alba A, Duran I (2017). Systemic treatment of renal cell cancer: A comprehensive review. Cancer Treat Re.

[8] Mlcochova H, Machackova T, Rabien A, Radova L, Fabian P, Iliev R (2016). Epithelial-mesenchymal transition-associated microRNA/mRNA signature is linked to metastasis and prognosis in clear-cell renal cell carcinoma. Sci Rep.

[9] Han C, Zhang C, Wang H, Li K, Zhao L (2021). Angiogenesis-related lncRNAs predict the prognosis signature of stomach adenocarcinoma. BMC Cancer.

[10] Kuczynski EA, Vermeulen PB, Pezzella F, Kerbel RS, Reynolds AR (2019). Vessel co-option in cancer. Nat Rev Clin Oncol.

[11] Gao S, Wang Y, Xu Y, Liu S (2023). An Angiogenesis-Related lncRNA Signature Is Associated with Prognosis and Tumor Immune Microenvironment in Breast Cancer. J Pers Med.

[12] Xing T, He H (2016). Epigenomics of clear cell renal cell carcinoma: mechanisms and potential use in molecular pathology. Chin J Cancer Res.

[13] Valdés-Mora F, Clark SJ (2015). Prostate cancer epigenetic biomarkers: next-generation technologies. Oncogene.

[14] Angulo JC, Manini C, López JI, Pueyo A, Colás B, Ropero S (2021). The Role of Epigenetics in the Progression of Clear Cell Renal Cell Carcinoma and the Basis for Future Epigenetic Treatments. Cancers (Basel).

[15] Peters I, Gebauer K, Dubrowinskaja N, Atschekzei F, Kramer MW, Hennenlotter J (2014). GATA5 CpG island hypermethylation is an independent predictor for poor clinical outcome in renal cell carcinoma. Oncol Rep.

[16] Petillo D, Kort EJ, Anema J, Furge KA, Yang XJ, Teh BT (2009). MicroRNA profiling of human kidney cancer subtypes. Int J Oncol.

[17] Teixeira AL, Ferreira M, Silva J, Gomes M, Dias F, Santos JI (2014). Higher circulating expression levels of miR-221 associated with poor overall survival in renal cell carcinoma patients. Tumour Biol.

[18] Zhang T, Xia W, Song X, Mao Q, Huang X, Chen B (2022). Super-enhancer hijacking LINC01977 promotes malignancy of early-stage lung adenocarcinoma addicted to the canonical TGF-β/SMAD3 pathway. J Hematol Oncol.

[19] Nojima T, Proudfoot NJ (2022). Mechanisms of lncRNA biogenesis as revealed by nascent transcriptomics. Nat Rev Mol Cell Biol.

[20] Tan YT, Lin JF, Li T, Li JJ, Xu RH, Ju HQ (2021). LncRNA-mediated posttranslational modifications and reprogramming of energy metabolism in cancer. Cancer Commun (Lond).

[21] Herman AB, Tsitsipatis D, Gorospe M (2022). Integrated lncRNA function upon genomic and epigenomic regulation. Mol Cell.

[22] Mimura I, Nangaku M (2023). Epigenetic regulation of angiogenesis and ischemic response by long noncoding RNA LEENE in diabetes. Kidney Int.

[23] Boos F, Oo JA, Warwick T, Günther S, Izquierdo Ponce J, Lopez M (2023). The endothelial-enriched lncRNA LINC00607 mediates angiogenic function. Basic Res Cardiol.

[24] Li X, Kuang Q, Peng M, Yang K, Luo P (2023). Basement Membrane-Associated lncRNA Risk Model Predicts Prognosis and Guides Clinical Treatment in Clear Cell Renal Cell Carcinoma. Biomedicines.

[25] Xu Z, Peng B, Liang Q, Chen X, Cai Y, Zeng S (2021). Construction of a ferroptosis-related nine-lncRNA signature for predicting prognosis and immune response in hepatocellular carcinoma. Front Immunol.

[26] Bai Y, Lin H, Chen J, Wu Y, Yu S (2021). Identification of prognostic glycolysis-related lncRNA signature in tumor immune microenvironment of hepatocellular carcinoma. Front Mol Biosci.

[27] Pang Y, Wang Y, Zhou X, Ni Z, Chen W, Liu Y (2023). Cuproptosis-Related LncRNA-Based Prediction of the Prognosis and Immunotherapy Response in Papillary Renal Cell Carcinoma. Int J Mol Sci.

[28] Lai J, Miao S, Ran L (2023). Ferroptosis-associated lncRNA prognostic signature predicts prognosis and immune response in clear cell renal cell carcinoma. Sci Rep.

[29] Deng Y, Guo K, Tang Z, Feng Y, Cai S, Ye J (2022). Identification and experimental validation of a tumor-infiltrating lymphocytes-related long noncoding RNA signature for prognosis of clear cell renal cell carcinoma. Front Immunol.

[30] Wang YW, Liu C, Chen YD, Yang B, Chen X, Ma G (2023). An angiogenesis-related lncRNA signature predicts the immune microenvironment and prognosis of breast cancer. Aging (Albany NY).

[31] Comandone A, Vana F, Comandone T, Tucci M (2021). Antiangiogenic Therapy in Clear Cell Renal Carcinoma (CCRC): Pharmacological Basis and Clinical Results. Cancers (Basel).

[32] Li JL, Harris AL (2009). Crosstalk of VEGF and Notch pathways in tumour angiogenesis: therapeutic implications. Front Biosci (Landmark Ed).

[33] Harrington LS, Sainson RC, Williams CK, Taylor JM, Shi W, Li JL (2008). Regulation of multiple angiogenic pathways by Dll4 and Notch in human umbilical vein endothelial cells. Microvasc Res.

[34] Babina IS, Turner NC (2017). Advances and challenges in targeting FGFR signalling in cancer. Nat Rev Cancer.

[35] Butti R, Das S, Gunasekaran VP, Yadav AS, Kumar D, Kundu GC (2018). Receptor tyrosine kinases (RTKs) in breast cancer: signaling, therapeutic implications and challenges. Mol Cancer.

[36] Yang OC, Maxwell PH, Pollard PJ (2013). Renal cell carcinoma: translational aspects of metabolism and therapeutic consequences. Kidney Int.

[37] Lei D, Chen Y, Zhou Y, Hu G, Luo F (2021). An angiogenesis-related long noncoding RNA signature correlates with prognosis in patients with hepatocellular carcinoma. Biosci Rep.

[38] Gong Q, Huang X, Chen X, Zhang L, Zhou C, Li S (2023). Construction and validation of an angiogenesis-related lncRNA prognostic model in lung adenocarcinoma. Front Genet.

[39] Sun L, Xi S, Zhou Z, Zhang F, Hu P, Cui Y (2022). Elevated expression of RIT1 hyperactivates RAS/MAPK signal and sensitizes hepatocellular carcinoma to combined treatment with sorafenib and AKT inhibitor. Oncogene.

[40] Hu X, Zhou X, Zhang J, Li L (2022). Sphingolipid metabolism is associated with osteosarcoma metastasis and prognosis: Evidence from interaction analysis. Front Endocrinol (Lausanne).

[41] Zhang Y, Li L, Tu Y, Feng Z, Li Z, Cao Y (2022). A DCS-related lncRNA signature predicts the prognosis and chemotherapeutic response of patients with gastric cancer. Biosci Rep.

[42] Wang C, Yang Y, Yin L, Wei N, Hong T, Sun Z (2020). Novel Potential Biomarkers Associated With Epithelial to Mesenchymal Transition and Bladder Cancer Prognosis Identified by Integrated Bioinformatic Analysis. Front Oncol.

